# Leisure-time physical activity and prevalence of non-communicable pathologies and prescription medication in Spain

**DOI:** 10.1371/journal.pone.0191542

**Published:** 2018-01-19

**Authors:** Pablo Fernandez-Navarro, María Teresa Aragones, Victoria Ley

**Affiliations:** 1 Cancer and Environmental Epidemiology Unit, National Center for Epidemiology, Carlos III Institute of Health, Madrid, Spain; 2 Consortium for Biomedical Research in Epidemiology & Public Health (CIBER en Epidemiología y Salud Pública - CIBERESP), Madrid, Spain; 3 Department of Sports and Health, Spanish Agency for Health Protection in Sports, National Sports Council, Madrid, Spain; Florida International University Herbert Wertheim College of Medicine, UNITED STATES

## Abstract

Our aims were to describe physical activity (PA) behaviour in Spain and to examine its association with the prevalence of some of the major non-communicable diseases and with the use of prescription medication. Individualized secondary data retrieved from the 2014 European Health Interview Survey (EHIS) for Spain were used to conduct a cross-sectional epidemiological study (n = 18926). PA was assessed by two different measures: a specific designed variable for EHIS and a leisure time PA frequency-based query of the national survey. Diseases analyzed were hypertension, diabetes, hypercholesterolemia, depression and anxiety. The use of prescription medication was also included in the study. Weighted percentages were computed and contingency tables were calculated to describe PA by levels of the traits and sociodemographic characteristics. Chi-square test was used to compare percentages between groups and weighted logistic regression models were used to assess the relationship between PA and the prevalence of the disease. About 73% of the Spanish population performs no PA at all or only occasionally during their leisure time, and only one third meets minimum PA international guidelines (≥ 150min/week). Men are considerably more active than women and less PA is observed as the education level decreases and as age increases. The risk of the diseases evaluated was up to three times higher among inactive individuals. This study provides national population-based estimations highlighting the impact of PA in Spain, not only in the prevalence of some of the major non-communicable diseases but also in reducing prescription medication, and the potential sex and socioeconomic influence.

## Introduction

Physical activity (PA) has important health benefits and can reduce the risk of some of the major non-communicable diseases such as diabetes, hypertension, obesity, depression, and musculoskeletal problems (reviewed in[1–5]). Moreover, the impact of PA on life expectancy has been determined in several studies, with an estimated up to 7 years gain in longevity and a reduction in premature mortality by 20–40%[6–8].

From an socioeconomic perspective, the estimated direct and indirect cost of inactivity in the EU-28 exceeds €80 billion per annum, including public health costs, prescription medications, functional limitations, disabilities, and loss of independence, as well as the loss of thousands of working hours and low productivity[9,10]. Furthermore, epidemiological studies show that aerobic exercise is inversely related to illicit drug use and abuse[11], which is a major public health problem that impacts society on multiple levels.

A worrying proportion of the world´s population fails to achieve the minimum PA levels recommended by the World Health Organization (WHO), which is ≥150 min/week, or declares to have a sedentary behaviour[12,13]. Although it is well known that physical inactivity is a leading risk factor for non-communicable diseases, there has been insufficient progress in advancing policies on this issue in many countries. Moreover, there is a lack of national population-based estimations of the association between PA and the aforementioned diseases. In this regard, internationally standardized surveys like the European Health Interview Survey (EHIS)[14] are a reliable tool for national surveillance studies and can be compared with those of other European countries. Politicians and stakeholders need compelling real data and useful tools to devote resources to design and implement public policies targeting populations of different characteristics, for example focusing on the less educated population, women and aged people. In this context, the aims of this study were to describe the PA behaviour in the Spanish population and to examine whether PA was associated with the prevalence of some of the major non-communicable diseases (hypertension, hypercholesterolemia, diabetes, depression and anxiety) and with the use of prescription medication.

## Materials and methods

### Study design and participants

Individualized secondary data retrieved from the 2014 EHIS for Spain was used to conduct a nationwide, descriptive, cross-sectional epidemiological study on PA.

The EHIS is a health information system conducted in the European Statistical System under the responsibility of Eurostat that uses comprehensive and coordinated surveys [14]. All European Union States share common guidelines for the survey modules (health determinants, health status, health care, background variables) and designs and based on a common questionnaire. The EHIS variables are stipulated in the EHIS Commission regulation (EU) No 141/2013. The methodological manual containing an example questionnaire, conceptual guidelines and interviewer instructions is available at the Eurostat website, as well as details on the EHIS methodology [15]. Data are collected using national questionnaires, which may sometimes comprise more questions than the EHIS. In Spain, the EHIS is conducted by the National Statistics Institute in collaboration with the Spanish Ministry of Health and Social Affairs. The survey is a computer-aided home-based personal interview including a nation-wide representative sample of civilian, non-institutionalized population aged ≥15 years and residing in primary family dwellings (households). Study subjects were selected by means of probabilistic multistage sampling, with the first-stage units being census sections and the second-stage units being primary family dwellings. The initial sample consisted of 37,500 households distributed in 2,500 sections selected by means of probabilistic multistage sampling, with the first-stage units being census sections and the second-stage units being primary family dwellings. In each household, an adult was randomly selected (using the Kish table) to respond to the survey. The Spanish survey includes interviews from 22,842 people with an estimated total error of ±1.01%. The data collection period was from January 2014 to February 2015.

Because the WHO recommendations for PA are designed for adults (≥18 years), subjects under the age of 18 were excluded from the analyses. Likewise, due to the difficulty of analyzing their PA performance and the high prevalence of diseases and treatments, the population over 74 years was also excluded; as such, the final population analyzed was n = 18,926.

### Physical activity

EHIS leisure time PA data are collected using national questionnaires, which may contain supplementary questions in addition to those contained in the EHIS basic common questionnaire. In Spain, PA is assessed using two variables: *(i)* the AerobePAR indicator, which is mandatory in the EHIS for all countries and *(ii)* the LPTA, an indicator of leisure time PA complementary to AerobePAR that is included in the Spanish questionnaire after agreement with the European Council.

To assess the total sports, fitness and recreational activity corresponding to the aerobic PA definition as recommended by WHO[16], we used the AerobePAR indicator, that includes frequency and intensity of PA, allowing semi-quantitative PA determination, according to the guidelines of the EHIS-PAQ[14]. This variable is estimated by adding the time of PA declared in the answers to the following two questions: 1) “How much time in total do you spend on sports, fitness or recreational (leisure) physical activities (at least 10 minutes continuously) in a typical week? and 2) “In a typical week, on how many days do you bicycle for at least 10 minutes continuously to get to and from places? And how much time do you spend bicycling in order to get to and from places on a typical day?” This variable has two levels: “<150 min of PA/week” and “≥150 min of PA/week”.

Second, the leisure time PA (LTPA) behaviour was assessed using a single item included in the national survey asking: "What is the answer that best describes your physical or sport activity in your leisure time?" a) I do not exercise at all; b) I perform PA or sports occasionally (walking or cycling, gardening, light gym, recreational activities that require a slight effort, etc.); c) I perform PA several times per month (sports, gymnastics, running, swimming, cycling, team games, etc.); and d) I perform sport training several times a week. We classified the population in four levels, 0, I, II and III according to their responses a), b), c) and d), respectively. This variable has been continued in the Spanish national survey since 2006, and therefore has an added value by allowing the analysis of historical trends.

### Sociodemographic characteristics and body mass index

The sociodemographic characteristics assessed were age, sex and education level, categorized as basic (up to primary school), secondary (secondary school or intermediate professional qualifications) and university (university or superior professional qualifications). The Body Mass Index (BMI) is defined as the weight in kilograms divided by the square of the height in meters, both self-reported by respondents during the survey[14]. Categories of body mass index (BMI), <25 kg·m^−2^, 25–29.9 kg·m^−2^ and ≥30 kg·m^−2^, were considered as 'under/normal weight', 'overweight' and 'obese', respectively, according to WHO guidelines[17].

### Non-communicable diseases and use of prescription medication

The five non-communicable diseases that we analyzed (hypertension, diabetes, hypercholesterolemia, depression and anxiety) are those for which there is stronger evidence on PA benefits [2,3,5] and each has a specific indicator in EHIS that can be analyzed unequivocally. Similarly, the covariates sex, age, BMI and education level were included as they are common factors that have shown to influence the prevalence and risk of non-communicable diseases and therefore could confound associations[4,18].

The use of prescription medication was assessed using the following (yes or no) question: “During the past two weeks, have you used any medicines that were prescribed for you by a doctor?”

### Statistical analysis

A descriptive analysis of PA (assessed using the two variables described) by sociodemographic characteristics, non-communicable diseases and prescription drug use was performed. To do this, weighted percentages and their 95% confidence intervals were computed and contingency tables were calculated. Chi square test of Independence was used for testing relationships between PA and the variables described before. Moreover, weighted logistic regression models adjusted for sex, age, BMI and education level were used to assess the relationship between PA and the prevalence of the diseases and between PA and drug consumption. All analyses were also performed stratified by sex, and in order to statistically contrast the possible interaction between PA and sex, the same logistic regression models described beforehand were performed including an interaction term. Analysis was performed using R software and its libraries "rmeta” and “survey”[19,20], adjusting for the design effects of the EHIS.

## Results

The sample used in this study comprised 18,926 participants with ages between 18 and 74 years, corresponding to 82.9% of the total sample (n = 22,842).

### Physical activity

The weighted percentages of subjects by each of the two PA variables assessed are shown in Table 1. According to the results, 34.4% of the Spanish population aged 18 to 74 years reported to perform no PA at all in their leisure time (level 0 of LTPA variable) and 38.9% reported to perform PA occasionally (level I). Likewise, only 33.24% of the population reported to engage in at least 150 min/week of PA in their leisure time according to the AerobePAR variable. The weighted percentages of individuals by LTPA level in each of the levels of AerobePAR are shown in S1 Fig. The population with a level of AerobePAR “<150 min/week” performed PA mainly at levels 0 and I according to the LTPA variable. Furthermore, the population with a level of AerobePAR “≥150 min/week” hardly ever performed PA of level 0 according to the LTPA variable.

**Table 1:** Weighted percentages (95%CI) of subjects by each of the two physical activities variables assessed (LTPA and AerobePAR) in each of the levels of BMI and sociodemographic variables assessed.

			Physical Activity (LTPA^a^)					Physical Activity (AerobePAR^b^)	
	N	%	0	I	II	III	p.value^c^	≥150 min	p.value^c^
**Total**	18926	100	34.4 (33.7–35.3)	38.9 (38.0–39.7)	14.0 (12.8–14.0)	13.3 (12.7–13.9)		33.2 (32.4–34.1)	
**Men**	9053	49.8 (48.9–50.6)	30.0 (28.8–31.1)	36.8 (35.6–38.0)	16.7 (15.7–17.7)	16.6 (15.7–17.6)	<0.001	38.8 (37.6–40.1)	<0.001
**Women**	9873	50.2 (49.4–51.1)	38.9 (37.7–40.1)	41.0 (39.8–42.2)	10.1 (9.4–10.9)	10.0 (9.3–10.8)		27.7 (26.6–28.8)	
**Age**									
18–24	1093	9.3 (8.7–9.9)	28.4 (25.4–31.6)	25.9 (23.0–29.2)	22.8 (20.0–25.9)	22.8 (20.0–26.0)	<0.001	44.3 (40.1–47.9)	<0.001
25–34	2504	17.8 (17.1–18.6)	32.6 (30.5–34.8)	32.2 (30.0–34.4)	16.9 (15.2–18.7)	18.3 (16.6–20.2)		39.3 (37.0–41.6)	
35–44	4559	23.3 (22.6–24.0)	35.0 (33.3–36.6)	35.3 (33.7–37.0)	15.9 (14.7–17.1)	13.9 (12.8–15.1)		32.6 (31.0–34.2)	
45–54	4076	20.9 (20.2–21.6)	37.0 (35.2–38.8)	40.3 (38.5–42.2)	11.4 (10.3–12.6)	11.3 (10.2–12.5)		31.3 (29.6–33.1)	
55–64	3569	16.1 (15.5–16.7)	36.1 (34.2–38.1)	46.6 (44.6–48.6)	8.9 (7.8–10,0)	8.5 (7.4–9.6)		27.7 (26.0–29.5)	
65–74	3125	12.7 (12.2–13.2)	34.1 (32.1–36.1)	52.2 (50.1–54.2)	6.1 (5.1–7.1)	7.7 (6.7–8.9)		28.0 (26.1–29.9)	
**BMI**									
Underweight	352	2.3 (2.0–2.6)	36.1 (30.3–42.3)	29.0 (23.9–34.8)	16.9 (12.8–22.0)	18.0 (13.5–23.6)	<0.001	33.4 (27.7–39.6)	<0.001
Normalweight	8279	46.6 (45.7–47.5)	30.0 (28.8–31.3)	37.0 (35.7–38.2)	16.2 (15.2–17.2)	16.8 (15.9–17.9)		37.7 (36.4–39.0)	
Overweight	6650	34.8 (34.0–35.7)	33.8 (32.4–35.2)	41.1 (39.7–42.6)	13.3 (12.3–14.4)	11.8 (10.9–12.8)		34.2 (32.8–35.6)	
Obese	3048	16.3 (15.7–17.0)	46.1 (43.9–48.3)	41.0 (38.9–43.2)	6.4 (5.5–7.5)	6.5 (5.6–7.7)		21.8 (20.1–23.6)	
**Education**									
Basic	9159	47.7 (46.8–48.6)	41.2 (39.9–42.4)	41.5 (40.2–42.7)	9.1 (8.3–9.9)	8.3 (7.6–9.1)	<0.001	26.9 (25.8–28.1)	<0.001
Intermediate	4018	22.6 (21.8–23.3)	31.9 (30.2–33.7)	36.7 (34.9–38.6)	15.7 (14.4–17.2)	15.6 (14.3–17.1)		38.5 (36.6–40.4)	
Superior	5749	29.8 (29.0–30.6)	25.6 (24.3–27.0)	36.4 (34.9–37.9)	18.5 (17.4–19.8)	19.5 (18.3–20.8)		39.4 (37.9–40.9)	

^a^LTPA (leisure time physical activity): 0 = never, I = occasional, II = several times/month, III = several times/week;

^b^AerobePAR (aerobic physical activity) levels: <150 minutes of physical activity/week and ≥150 minutes of physical activity/week;

^c^ p.value = p value from Chi square test of Independence.

### Sociodemographic characteristics

Table 1 also shows that, irrespective of the variable of PA assessed, men reported to be more active than women and PA decreases with age; for example, the weighted percentage of subjects performing PA several times a month (level II of LTPA) was 22.8% in the 18–34 age group, 15.9% in the 35–44 age group and 6.1% for those aged 65–74 years. Similarly, the weighted percentage of individuals performing at least 150 min/week of PA (AerobePAR) decreased from 44.4% in the 18–34 age group to 32.6% in the 35–44 age group and was 28.0% for those aged 65–74 years.

Approximately 87% of obese individuals reported no PA at all or only occasionally (LTPA), and 78.2% reported less than 150 min of PA per week (AerobePAR).

Finally, the population with basic education reported lower PA levels, assessed using either of the two measures; only 17% of this population reported a PA of level II or III of LTPA and 26.9% at least 150 min of PA per week, compared with 38% and 40%, respectively, in the population with a higher education.

### Physical activity and non-communicable diseases

The prevalence of the five non-communicable diseases assessed by PA levels is shown in Table 2. Individuals who were more active were less likely to have any of the five diseases, and the prevalence of the diseases was significantly lower in the more active population measured by both LTPA and AerobePAR variables, than in the population that did not exercise at all. In relation to the LTPA variable, the prevalence in the active population was considerably lower for all diseases and risk factors analyzed; about 50% lower for hypercholesterolemia and hypertension and about 30% lower for diabetes, depression and anxiety. It is also noteworthy that disease prevalence was not notably lower in the population practicing the most intense level of PA, level III, than in level II. Similar results were observed assessing PA with the AerobePAR variable. Accordingly, the prevalence of hypercholesterolemia and hypertension were reduced by 30% and those of diabetes, depression and anxiety were reduced by approximately 50% in the population that reported to perform at least 150 min per week PA.

**Table 2:** Estimated prevalence (95%CI) of chronic diseases and use of prescription medication in Spanish population in relation with physical activity.

Trait			Physical Activity (LTPA^a^)					Physical Activity (AerobePAR^b^)	
	N	%	0	l	ll	lll	p.value^c^	≥150 min	p.value^c^
Hypercholesterolemia	3365	15.3 (14.7–15.9)	16.6 (15.5–17.7)	18.0 (17.1–19.0)	10.4 (9.1–11.8)	8.9 (7.8–10.3)	<0.001	12.4 (11.5–13.3)	<0.001
Diabetes	1178	5.5 (5.2–5.9)	7.31 (6.6–8.1)	6.4 (5.8–7.0)	2.00 (1.4–2.7)	2.0 (1.4–2.9)	<0.001	3.6 (3.1–4.2)	<0.001
Hypertension	3412	15.5 (14.9–16.1)	17.9 (16.9–19.0)	18.4 (17.5–19.5)	8.6 (7.5–9.9)	7.6 (6.5–8.9)	<0.001	11.8 (10.9–12.7)	<0.001
Depression	1558	7.3 (6.8–7.7)	10.9 (10.1–11.8)	7.0 (6.4–7.8)	3.0 (2.3–3.8)	2.9 (2.2–3.8)	<0.001	4.8 (4.2–5.5)	<0.001
Anxiety	1560	7.6 (7.2–8.1)	10.7 (9.8–11.6)	7.7 (7.0–8.5)	3.8 (3.0–4.8)	3.5 (2.7–4.5)	<0.001	4.9 (4.3–5.6)	<0.001
Use of prescription medication	10334	51.2 (50.4–52.1)	56.1 (54.6–57.6)	55.8 (54.4–57.2)	39.0 (36.7–41.4)	37.7 (35.3–40.0)	<0.001	45.5 (44.0–47.0)	<0.001

^a^LTPA (leisure time physical activity) levels: 0 = never, I = occasional, II = several times/month, III = several times/week.

^b^AerobePAR (aerobic physical activity) levels: <150 minutes of physical activity/week and ≥150 minutes of physical activity/week.

^c^p.value = p value from Chi square test of Independence.

### PA versus use of prescription medication

As shown in Table 2, those individuals reporting to be physically active were more likely to have a lower use of prescription medication. Results from the analysis of LTPA variable showed that the use of prescribed medications was approximately 20% lower in more active subjects than in inactive subjects. The use of medication in those individuals were similar in levels 0 and I, as well as in levels II and III. According to the AerobePAR variable, active subjects used 9% less medications than their inactive peers.

### Association analysis

**Fig 1:**
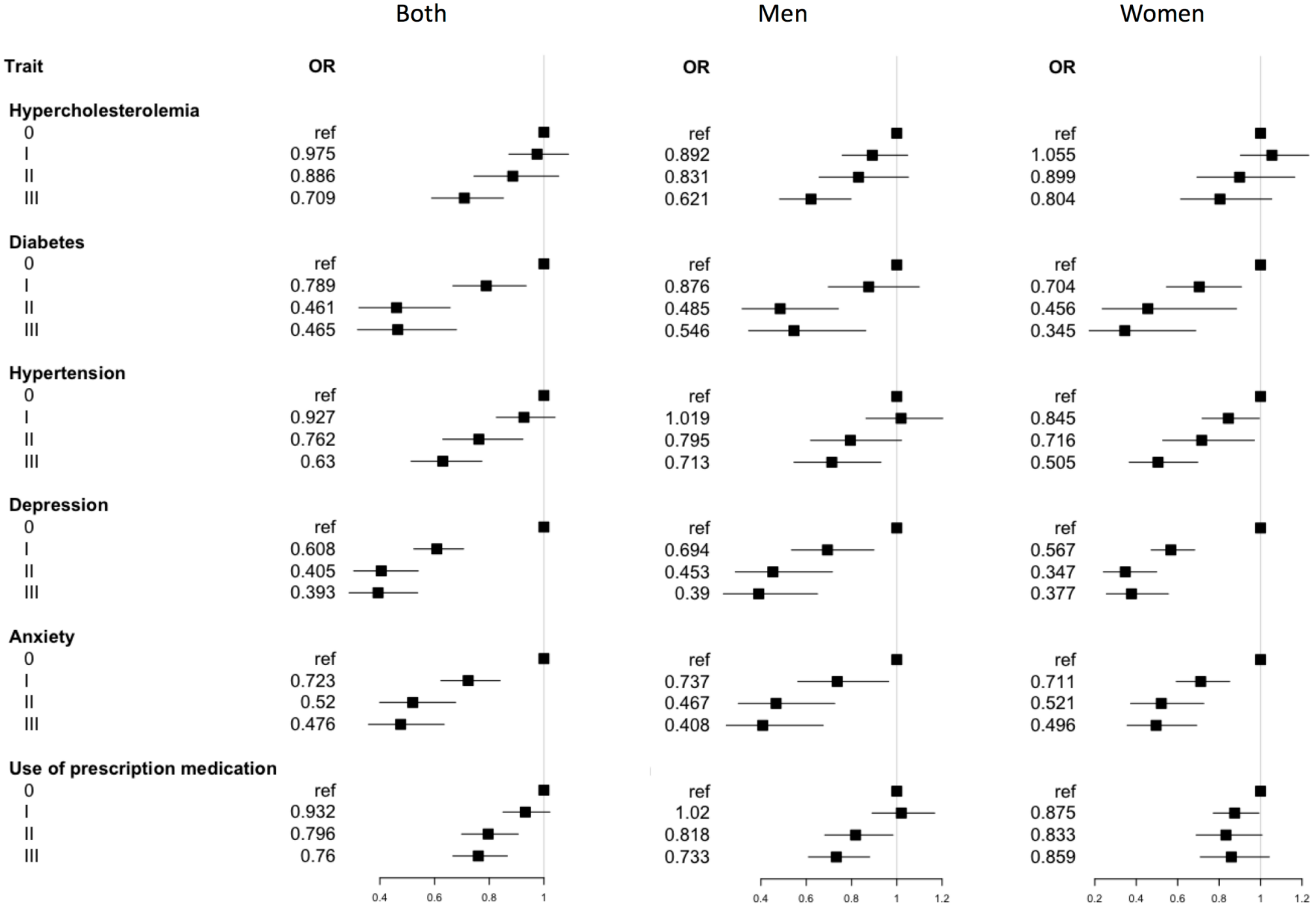
Forest plot of odds ratios and 95% confidence intervals for the association analysis between non-communicable diseases or use of prescription medication and physical activity (LTPA).

**Fig 2:**
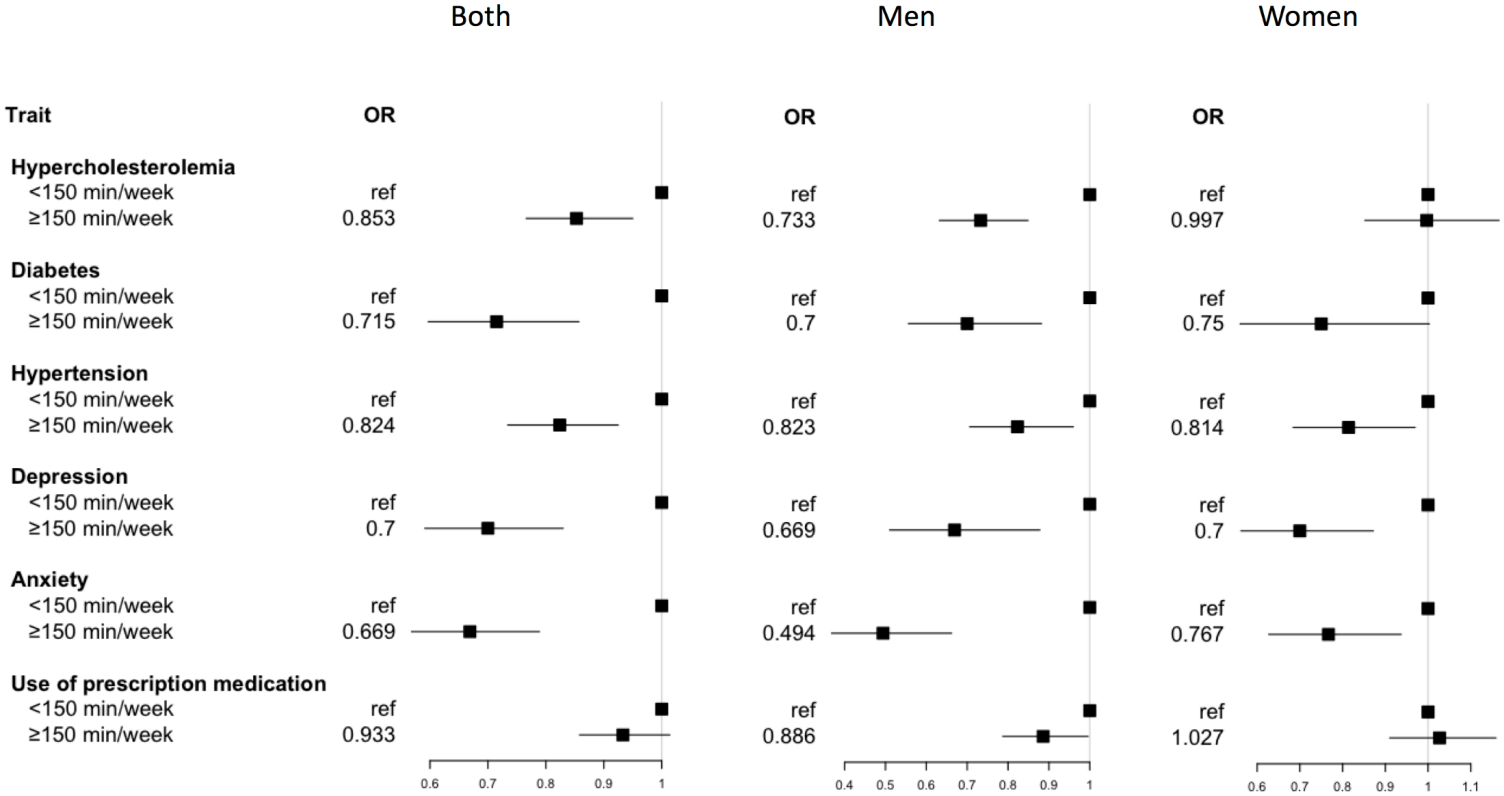
Forest plot of odds ratios and 95% confidence intervals for the association analysis between non-communicable diseases or use of prescription medication and physical activity (AerobePAR).

The results of the association analysis are shown in Figs 1 and 2. The risk of having any of the non-communicable diseases assessed was significantly reduced as the LTPA level increased, particularly in levels II and III: compared with the inactive participants, the odds ratio (OR) of level III was 0.70 (95%CI, 0.59–0.85) for hypercholesterolemia; 0.46 (95%CI, 0.32–0.68) for diabetes; 0.63 (95%CI, 0.51–0.77) for hypertension, 0.39 (95%CI, 0.29–0.54) for depression and 0.46 (95%CI, 0.36–0.64) for anxiety. Similar results were observed when the AerobePAR variable was assessed.

Although the impact of physical inactivity (PI) for the prevalence of these health variables was clear in men and women, the associations of PA with hypercholesterolemia and use of medication were stronger in men, whereas for level III LTPA, the association of PA with hypertension was stronger in women (see also S1 Table).

Regarding the use of prescription medication, there was a positive association between the two variables of PA (AerobePAR and LTPA) and this health indicator. The frequency of medication consumption in more active people (level III in LTPA variable) was approximately 25% lower than in inactive people. This association was stronger in men than in women, in which the association was found only when analyzing the LTPA variable (see also S1 Table).

## Discussion

Our study indicates that about 73% of the Spanish population performs no PA at all or only occasionally in their leisure time and only one third engage in at least 150 min/week. Moreover, men are considerably more active than women and PA levels decrease with age, lower education level, and higher BMI. Finally, the main results based on the adjusted association analysis between five major non-communicable diseases or the use of prescribed medication and PA show a higher risk of these diseases and the health indicator mentioned among those subjects who report low PA levels. When stratified by sex, the results indicate that in two of these diseases, diabetes and hypertension, the reduction of risks observed at high PA levels are stronger in women than in men.

Regarding PA behavior, in Spain, as in other European Mediterranean countries, the levels of PA are lower than in northern European countries. In addition, Spanish women are particularly inactive compared with those of other southern countries[12,21].

Although the validity of self-reported BMI for assessing obesity is arguable[22], we still considered it was important to include it in our analyses. Consistent with previous research[23,24], we found an inverse association of PA with obesity but not with overweight. However, it is not possible to infer a cause-effect relationship from this association, with obesity having maybe a reducing effect on PA, or PA having such effect on obesity, or both.

Regarding the socioeconomic variable studied here (*i*.*e*., education level), our findings are in line with other studies indicating that more educated population tends to practice more PA and sports[12,21]. The prevalence of PI in the population with a basic level of education or below was almost twice that in of those with a higher education. Low educational level accompanied by low economic status has also been associated with a higher prevalence of obesity[25] and therefore this population is of particular interest when designing health and education policies aiming at promoting PA, particularly in the most inactive people.

There is abundant epidemiological and clinical evidence showing that PA is associated with a reduction in all-cause mortality and in the prevalence of cardiovascular disease, hypertension, metabolic syndrome, type 2 diabetes and depression, among others[2,3,5,26]. Although the present work is an observational analysis of the association of PA with the prevalence of diseases and does not prove a causal relationship, the results are in line with these and other studies indicating that PA confers a considerable protection against diabetes, hypertension and hypercholesterolemia as well as against mental health problems such as anxiety and depression. The benefits of PA are also well exemplified in certain types of cancer. However, unfortunately, the EHIS survey includes only one question assessing cancer without specifying the type.

The results show that PA is strongly associated with a reduced risk of the diseases analyzed, particularly diabetes, depression and anxiety, in which the risk of having these diseases is reduced by up to 60% in active people. There was also a clear association of PA with a lower risk of hypertension (50% in woman and 30% in men), and with a lower prevalence of total hypercholesterolemia, especially in men (40%). These results are consistent with those describing the effect of PA in the prevention and treatment of diabetes[27,28], hypertension[29], in regulating the lipid profile[30], mental problems[31], as well as in a number of other diseases[3,32]. Also, our findings would be in line with studies showing that PA prevents hyperlipidemia and improves the lipid profile[30,33,34].

The results from the association analysis between PA and mental health diseases are in accord with a number of studies showing the effect of PA for the prevention and treatment of depression and anxiety, suggesting different physiological mechanisms that might be involved in this effect[3,32,35] and a strong association of the prevalence of these diseases with PI[36,37]. The causality of this association has also not been sufficiently demonstrated.

This study also showed a dose-response effect of PA, increasing the protective effect as the PA level increased. In the general population, the most efficient PA level to reduce disease prevalence was LPTA level II and little or no further improvement was observed in those at level III for diabetes, depression, or anxiety (see Table 2 and Fig 1). However, when analyzing groups by gender, there was a higher protective effect for diabetes and hypertension at level III in women. This effect was only seen when analyzing the LTPA indicator, which classifies the population in four groups of PA, and is therefore more sensitive. Nonetheless, except for hypercholesterolemia, the reduction of the risks is found from level I of LPTA, particularly in women. These observations support previous studies showing that even a slight increase in PA is effective for prevention of non-communicable diseases and reduction of mortality in the sedentary population[7,8,38].

The molecular mechanisms underlying the health benefits of different types of activities (i.e., involving more aerobic vs. more resistance exercises) are not yet fully identified. However, there are studies showing that not only aerobic, but also resistance exercise training has an important role in improving health parameters such as insulin sensitivity, muscle mass/function and age-related decline in physical function. It would be interesting to analyze the possible association of different exercise modalities and also of sedentary behavior (*i*.*e*., total daily sitting time), with the prevalence of non-communicable diseases and cardiovascular risk factors.

To strengthen our results, the associations between PA and non-communicable diseases or health indicators were analyzed using two variables: LTPA and AerobePAR. LTPA considers, but does not quantify, the frequency and intensity of leisure-time PA, and allows classification of the population into four categories of PA levels, increasing the sensitivity of the potential PA effects. To further assess the PA levels of the population, we used the variable AerobePAR, which includes quantitative factors such as frequency, intensity and duration of the types of PA queried. Indeed, this indicator of the EHIS-PAQ questionnaire estimates aerobic PA focusing on sports, fitness and recreational activities (leisure), which are the primary health-enhancing types of PA. AerobePar has been specifically developed to measure compliance with PA international guidelines, allowing to distinguish individuals who do ‘insufficient health-enhancing aerobic PA’ form those who engage in ‘sufficient health-enhancing aerobic PA’. [14].

Some potential limitations of the current analysis must be considered. PA is difficult to determine and quantify, especially in a survey with subjective responses. Likewise, several of the data obtained from the interviews were self-reported and may be subject to bias. Nevertheless, EHIS has been validated and designed considering these aspects and to minimize the effects of non-respondent or self-reported bias. An additional strength of our study comes from the fact that the EHIS variables (including AerobePar) have been designed for a multinational health interview survey context and validated in large-scale health interview surveys in Europe, where the survey is compulsory in all Member States since 2013 [14,15]. In addition, the large sample size, a randomly selected population, the employment of a standardized survey, and training of the data collectors, should minimize these limitations.

Regarding the analysis of chronic diseases, the purpose of the survey is to monitor the prevalence of specific chronic diseases or conditions. The question is whether the person has or had a specific chronic disease or condition in the past 12 months, and individuals living with these chronic conditions over several years are included in the positive (yes) group. Although this might represent a limitation of the analysis, from a public health perspective and for policy making it is the best alternative to determine the prevalence of chronic diseases using this type of surveys.

There are limitations inherent to the lack of quantitative determination of PA using the questionnaire, particularly with the semi-quantitative LTPA variable. However, the consistency of our findings across different diseases, and the fact that all regression models had good specifications and fit, should add further confidence to the study.

Regarding other indicators, the use of prescription medication does not specifically assess the medication in question that has been shown to be related to PA or to the consequences of a sedentary behaviour. The study would be enriched if medications were categorized into common indications (*e*.*g*., antihypertensive, lipid-lowering, antidiabetic). Unfortunately, the EHIS survey includes only one question without specifying the type of medicament. Nevertheless, it is a generic indicator that is useful to assess the population heath status and the potential costs to the health system.

Another limitation is the cross-sectional design of the analysis, which does not allow us to establish causal relationships. Nevertheless, there is abundant evidence showing the effect of PI as the origin of these and other diseases. In addition, while questionnaires are most frequently used in epidemiological and cross-sectional studies, they may have bias of over- or under-estimation of the self-reported data.

There is a worldwide trend towards less PA and our study shows that Spain has a worryingly low prevalence of sedentary behaviour, with 73% of the population doing either no physical activity at all or just very little PA during their leisure time. Although it is well known that PI is one of the four leading risk factors for non-communicable diseases, there has been insufficient progress in many countries regarding policies to combat PI [39]. The evidence for effectiveness of PA interventions has been shown in numerous scientific studies, but these interventions have not been scaled up to the population level. Analysis of the harmonized EHIS data adds quality and comparability on health information in the EU countries, allowing for a better design and testing of public policies.

Governments need to undertake effective interventions, which should be based on scientific evidence, policy monitoring, and cross-sector collaboration. These policies would benefit from being tailored to specific groups. Our study shows that *(i)* there is a strong association between PA and some of the most common diseases, as well as an indicator of health cost as is prescription of medications, and *(ii)* there is a demographic influence (level of education, age and gender) on such associations. Therefore, our results support the need to design more effective interventions, focusing on specific sectors such as women, less educated population, or people over 50 years old.

Finally, from a socioeconomic point of view, PI has an enormous impact and according to a recent publication, PI represents a cost of € 990 million in Spain for its impact on cardiovascular disease, diabetes, colon and breast cancer and € 5,000 million related to work productivity, tourism, crime, education, or mental health problems[10]. While difficult to determine, the impact on the functional dependence and disabilities of the elderly, drug spending and healthy life years is also important.

## Conclusions

The results of this study provide national population-based estimations highlighting the impact of PI in Spain not only for the prevalence of some of the major non-communicable diseases, but also for reducing prescription medications, and the potential influence of sex and level of education. Reducing PI may not only have a great impact on decreasing the risk of many non-communicable diseases and mortality, but also on many social and health problems related to functional dependence and disability of the elderly, who will have a longer healthy life. These data may serve to implement preventive measures and make more effective policies promoting physical activity and sport.

## Supporting information

S1 Fig Weighted percentage of individuals by level of AerobePAR variable in each of the levels of the LTPA variable. The confidence intervals for the percentages are represented by vertical lines.
(TIFF)

S1 Table P value for the interaction term between physical activity and sex in the logistic regression models for the association analysis between non-communicable diseases or use of prescription medication and physical activity (AerobePAR and LTPA). p.value: p values of the contrast for the interaction between physical activity and sex in the logistic regression models.
(DOCX)
