# Supplementary figures and images for: Leisure-time physical activity and prevalence of non-communicable pathologies and prescription medication in Spain
Source: PLoS One. 2018 Jan 19;13(1):e0191542. doi: 10.1371/journal.pone.0191542 (PMC5774808; doi:10.1371/journal.pone.0191542)

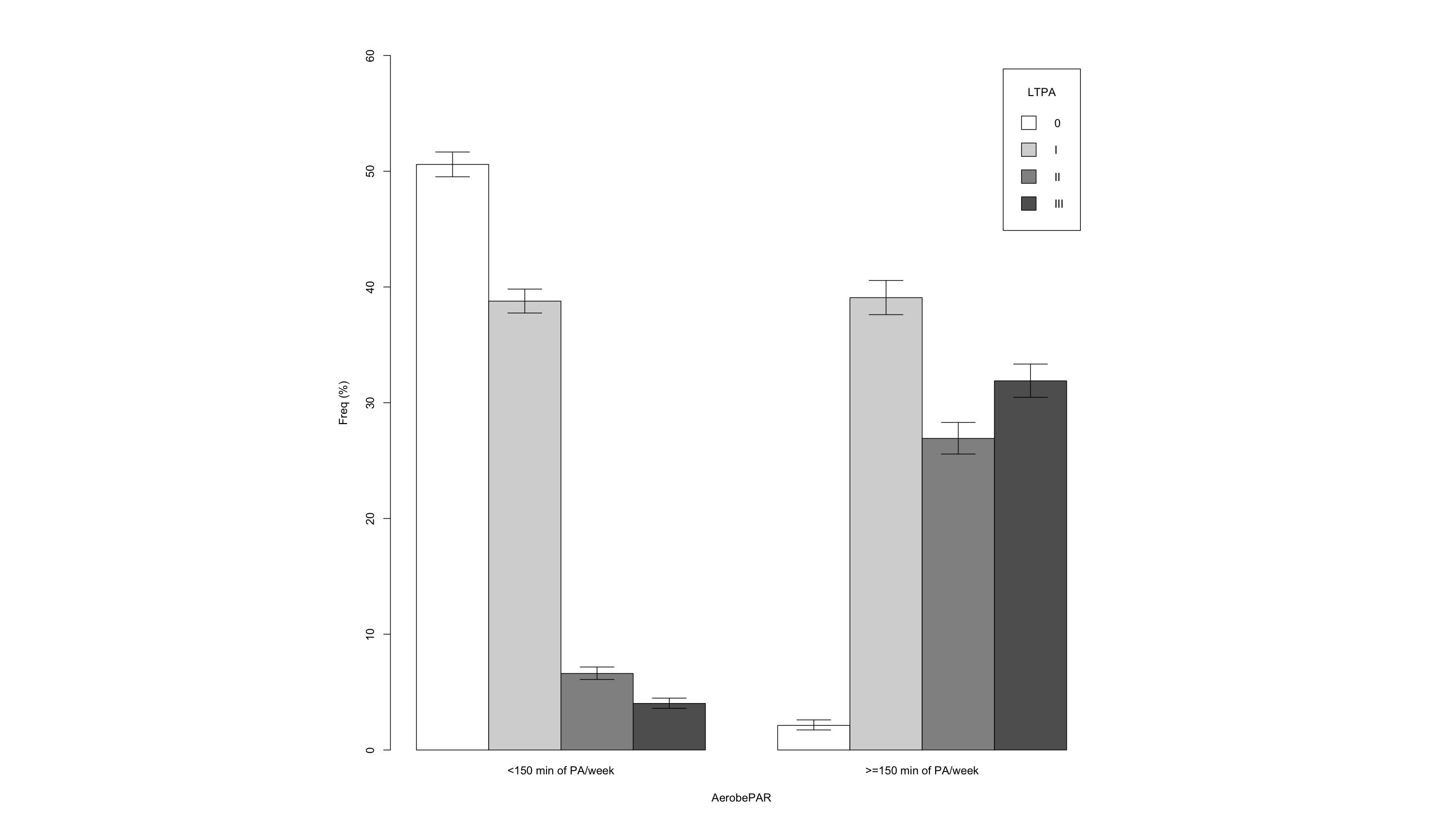

Supplement: S1 Fig — The confidence intervals for the percentages are represented by vertical lines. (TIFF) [file pone.0191542.s001.tiff]
