# Supplementary material for: Leisure-time physical activity and prevalence of non-communicable pathologies and prescription medication in Spain
Source: PLoS One. 2018 Jan 19;13(1):e0191542. doi: 10.1371/journal.pone.0191542 (PMC5774808; doi:10.1371/journal.pone.0191542)
Supplement: S1 Table — p.value: p values of the contrast for the interaction between physical activity and sex in the logistic regression models. (DOCX) [file pone.0191542.s002.docx]

| **Trait** | **Physical Activity** | | **p.value** |
| --- | --- | --- | --- |
| Hypercholesterolemia | LTPA | 0 | ref |
|  |  | I | 0.080 |
|  |  | II | 0.853 |
|  |  | III | 0.212 |
|  | AerobePAR | <150 min/week | ref |
|  |  | ≥150 min/week | 0.006 |
| Diabetes | LTPA | 0 | ref |
|  |  | I | 0.102 |
|  |  | II | 0.717 |
|  |  | III | 0.181 |
|  | AerobePAR | <150 min/week | ref |
|  |  | ≥150 min/week | 0.956 |
| Hypertension | LTPA | 0 | ref |
|  |  | I | 0.093 |
|  |  | II | 0.279 |
|  |  | III | 0.032 |
|  | AerobePAR | <150 min/week | ref |
|  |  | ≥150 min/week | 0.618 |
| Depression | LTPA | 0 | ref |
|  |  | I | 0.295 |
|  |  | II | 0.163 |
|  |  | III | 0.603 |
|  | AerobePAR | <150 min/week | ref |
|  |  | ≥150 min/week | 0.911 |
| Anxiety | LTPA | 0 | ref |
|  |  | I | 0.903 |
|  |  | II | 0.928 |
|  |  | III | 0.816 |
|  | AerobePAR | <150 min/week | ref |
|  |  | ≥150 min/week | 0.035 |
| Use of prescription medication | LTPA | 0 | ref |
|  |  | I | 0.020 |
|  |  | II | 0.838 |
|  |  | III | 0.244 |
|  | AerobePAR | <150 min/week | ref |
|  |  | ≥150 min/week | 0.050 |
